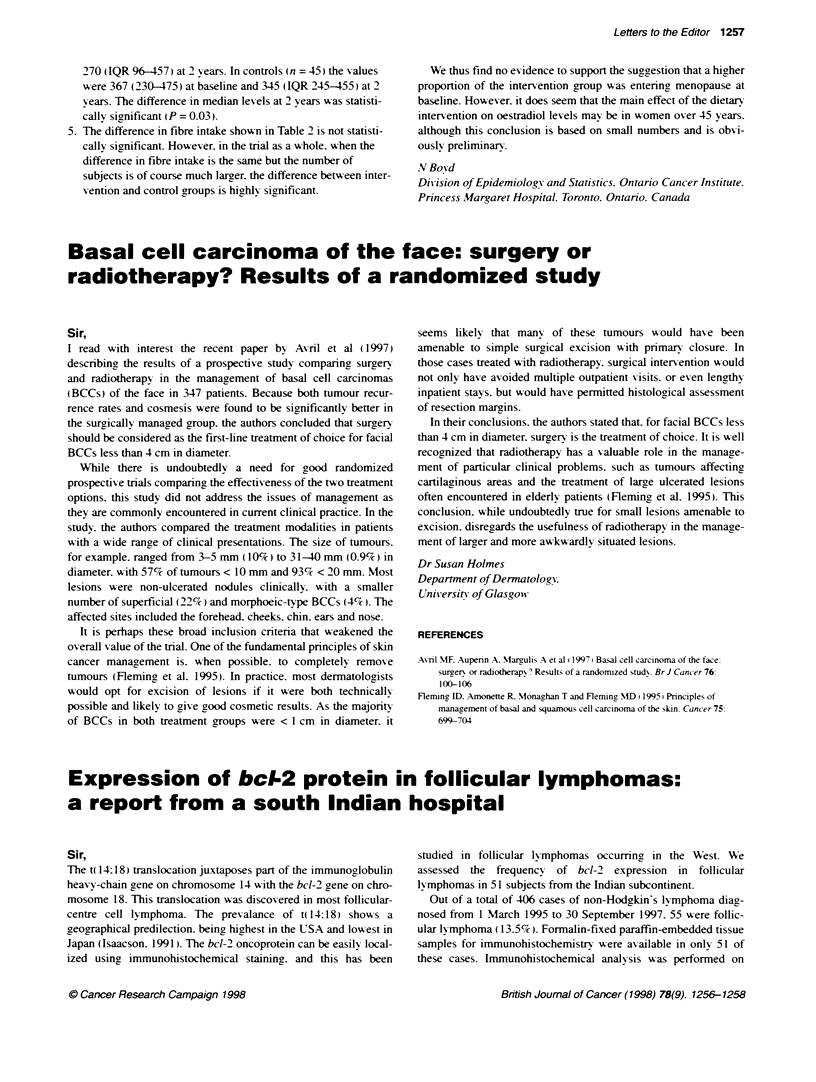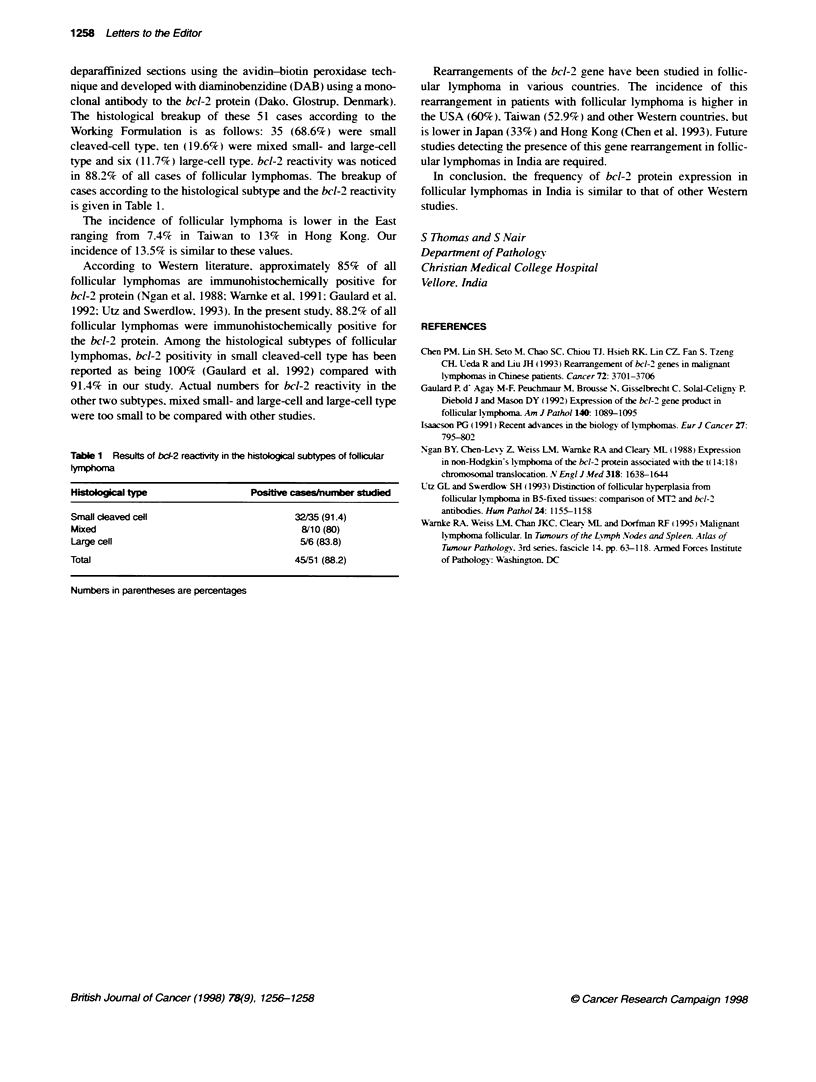# Expression of bcl-2 protein in follicular lymphomas: a report from a south Indian hospital.

**DOI:** 10.1038/bjc.1998.667

**Published:** 1998-11

**Authors:** S. Thomas, S. Nair


### Expression of *bcl*-2 protein in follicular lymphomas: a report from a south Indian hospital

**Sir,**

The t(14;18) translocation juxtaposes part of the immunoglobulin heavy-chain gene on chromosome 14 with the *bcl*-2 gene on chromosome 18. This translocation was discovered in most follicular-centre cell lymphoma. The prevalance of t(14;18) shows a geographical predilection, being highest in the USA and lowest in Japan (Isaacson, 1991). The *bcl*-2 oncoprotein can be easily localized using immunohistochemical staining, and this has been studied in follicular lymphomas occurring in the West. We assessed the frequency of *bcl*-2 expression in follicular lymphomas in 51 subjects from the Indian subcontinent.

Out of a total of 406 cases of non-Hodgkin's lymphoma diagnosed from 1 March 1995 to 30 September 1997, 55 were follicular lymphoma (13.5%). Formalin-fixed paraffin-embedded tissue samples for immunohistochemistry were available in only 51 of these cases. Immunohistochemical analysis was performed on

© Cancer Research Campaign 1998

deparaffinized sections using the avidin–biotin peroxidase technique and developed with diaminobenzidine (DAB) using a monoclonal antibody to the *bcl*-2 protein (Dako, Glostrup, Denmark). The histological breakup of these 51 cases according to the Working Formulation is as follows: 35 (68.6%) were small cleaved-cell type, ten (19.6%) were mixed small- and large-cell type and six (11.7%) large-cell type. *bcl*-2 reactivity was noticed in 88.2% of all cases of follicular lymphomas. The breakup of cases according to the histological subtype and the *bcl*-2 reactivity is given in Table 1.

The incidence of follicular lymphoma is lower in the East ranging from 7.4% in Taiwan to 13% in Hong Kong. Our incidence of 13.5% is similar to these values.

According to Western literature, approximately 85% of all follicular lymphomas are immunohistochemically positive for *bcl*-2 protein (Ngan et al, 1988; Warnke et al, 1991; Gaulard et al, 1992; Utz and Swerdlow, 1993). In the present study, 88.2% of all follicular lymphomas were immunohistochemically positive for the *bcl*-2 protein. Among the histological subtypes of follicular lymphomas, *bcl*-2 positivity in small cleaved-cell type has been reported as being 100% (Gaulard et al, 1992) compared with 91.4% in our study. Actual numbers for *bcl*-2 reactivity in the other two subtypes, mixed small- and large-cell and large-cell type were too small to be compared with other studies.

**Table 1** Results of *bcl*-2 reactivity in the histological subtypes of follicular lymphoma

| Histological type | Positive cases/number studied |
|---|---|
| Small cleaved cell | 32/35 (91.4) |
| Mixed | 8/10 (80) |
| Large cell | 5/6 (83.8) |
| Total | 45/51 (88.2) |

Numbers in parentheses are percentages

Rearrangements of the *bcl*-2 gene have been studied in follicular lymphoma in various countries. The incidence of this rearrangement in patients with follicular lymphoma is higher in the USA (60%), Taiwan (52.9%) and other Western countries, but is lower in Japan (33%) and Hong Kong (Chen et al, 1993). Future studies detecting the presence of this gene rearrangement in follicular lymphomas in India are required.

In conclusion, the frequency of *bcl*-2 protein expression in follicular lymphomas in India is similar to that of other Western studies.

*S Thomas and S Nair*
*Department of Pathology*
*Christian Medical College Hospital*
*Vellore, India*

### REFERENCES

Chen PM, Lin SH, Seto M, Chao SC, Chiou TJ, Hsieh RK, Lin CZ, Fan S, Tzeng CH, Ueda R and Liu JH (1993) Rearrangement of *bcl*-2 genes in malignant lymphomas in Chinese patients. *Cancer* **72**: 3701–3706

Gaulard P, d' Agay M-F, Peuchmaur M, Brousse N, Gisselbrecht C, Solal-Celigny P, Diebold J and Mason DY (1992) Expression of the *bcl*-2 gene product in follicular lymphoma. *Am J Pathol* **140**: 1089–1095

Isaacson PG (1991) Recent advances in the biology of lymphomas. *Eur J Cancer* **27**: 795–802

Ngan BY, Chen-Levy Z, Weiss LM, Warnke RA and Cleary ML (1988) Expression in non-Hodgkin's lymphoma of the *bcl*-2 protein associated with the t(14;18) chromosomal translocation. *N Engl J Med* **318**: 1638–1644

Utz GL and Swerdlow SH (1993) Distinction of follicular hyperplasia from follicular lymphoma in B5-fixed tissues: comparison of MT2 and *bcl*-2 antibodies. *Hum Pathol* **24**: 1155–1158

Warnke RA, Weiss LM, Chan JKC, Cleary ML and Dorfman RF (1995) Malignant lymphoma follicular. In *Tumours of the Lymph Nodes and Spleen. Atlas of Tumour Pathology*, 3rd series, fascicle 14, pp. 63–118. Armed Forces Institute of Pathology: Washington, DC

© Cancer Research Campaign 1998